# Sleep Deprivation and Subchronic Arsenite Exposure Synergistically Induced Skeletal Muscle Aging by Disrupting Melatonin and Cortisol Secretion in Mice

**DOI:** 10.3390/toxics13020097

**Published:** 2025-01-27

**Authors:** Hongyi Yang, Xingyu Chen, Xuanfeng Yu, Baofei Sun, Junyan Tao, Xiong Chen

**Affiliations:** 1 The Key Laboratory of Environmental Pollution Monitoring and Disease Control, Ministry of Education, School of Public Health, Department of Toxicology, Guizhou 561113, China; gmu_yhy@163.com (H.Y.); gmu_cxy@163.com (X.C.); yxf2931164955@163.com (X.Y.); sunbaofei@sina.com (B.S.); 2 Key Laboratory of Human Brain Bank for Functions and Diseases of Department of Education of Guizhou Province, Guizhou Medical University, Guian New Area, Guizhou 561113, China; 3 Collaborative Innovation Center for Prevention and Control of Endemic and Ethnic Regional Diseases Co-Constructed by the Province and Ministry, Guizhou Medical University, Guian New Area, Guizhou 561113, China

**Keywords:** sleep deprivation, arsenic, skeletal muscle aging, melatonin, cortisol

## Abstract

In recent years, the influence of environmental factors on organismal aging has garnered increasing attention. Studies have shown that sleep deprivation and environmental pollutants could accelerate the emergence of multiple organismal aging phenotypes. In addition, studies have shown that chronic exposure to sodium arsenite (iAs) induces skeletal muscle atrophy and the inhibition of melatonin secretion in rats. This study aimed to reveal the synergistic effect of sleep deprivation and arsenite exposure on skeletal muscle aging, including reduced limb grip strength and skeletal muscle mass, along with the serum levels of melatonin (MT) and cortisol (COR) in C57BL/6J mice. The results demonstrated that while exposure to arsenite for 12 weeks or sleep deprivation (SD) for 4 weeks did not exert significant effects on limb grip strength or skeletal muscle mass, their combination exhibited a synergistic effect on skeletal muscle aging. Notably, the iAs+SD group exhibited a significant decline in limb grip strength by Week 12, accompanied by a reduced gastrocnemius muscle mass and muscle index. The pathological analysis showed muscle fiber atrophy, a shift towards slow-twitch muscle fibers (type I), and shortened telomere length. Additionally, oxidative damage was increased in the SD and iAs+SD groups, with decreased levels of SOD and GPx and elevated levels of MDA in the iAs+SD group. The serum MT level and MT/COR ratio were significantly reduced, while the serum COR level was elevated in the iAs+SD group compared to the other groups. A correlation analysis further revealed that the serum MT level and the MT/COR ratio were positively correlated with limb grip strength, muscle index, and telomere length, whereas the serum COR level exhibited negative correlations with these parameters. These findings suggest that sleep deprivation and subchronic exposure to arsenite synergistically induce skeletal muscle aging, and that the disruption of the balance between MT and COR potentially serves as a significant risk factor.

## 1. Introduction

Arsenic, a well-known toxic metalloid, is widely distributed in contaminated drinking water [1]. Exposure to arsenic is a global public health issue, as approximately 200 million people around the world are exposed to arsenic at levels that pose a threat to health [1,2]. In groundwater, arsenic is present as trivalent arsenite [As(III)] while under oxidizing conditions, and as pentavalent arsenate [As(V)] while under reducing conditions, and arsenite [As(III)] can be modified by the body to a variety of As(III) and As(V) species. Long-term exposure to arsenic could induce multi-organ and multi-system damage, including damage to the liver, lungs, nervous system, and cardiovascular system [3]. In addition, epidemiological studies have revealed that arsenic exposure is associated with reduced skeletal muscle mass [4,5]. In an in vivo study, arsenic trioxide was found to induce autophagy and insulin resistance in skeletal muscle, leading to a decline in skeletal muscle mass [6].

Sleep is essential for maintaining a healthy lifestyle, as it promotes organismal repair and improves cognitive function and emotional stability [7,8]. In recent years, the acceleration of social rhythms and changes in people’s lifestyles have reduced the amount of sleep that individuals receive, thus becoming a global health issue. Studies have shown that an insufficient amount of sleep is associated with various adverse health outcomes, including metabolic disease, cardiovascular diseases, and sarcopenia [9,10]. Sleep loss could restrain peripheral immune function while promoting the accumulation of reactive oxygen species (ROS) and disrupting the metabolic functions of multiple organs, including skeletal muscle.

Skeletal muscle is the largest organ in the human body, accounting for approximately 40% of body mass. In addition to its role in locomotion, skeletal muscle plays a vital role in energy metabolism, glucose homeostasis, and endocrine regulation, highlighting its physiological significance. In recent years, sleep quality has been associated with the health of skeletal muscle. Numerous epidemiological studies have suggested that adequate sleep is required for the maintenance of skeletal muscle mass [11,12]. Chronic sleep loss could increase the breakdown of muscle proteins, leading to muscle atrophy [12]. However, it remains unclear whether sleep deprivation could act synergistically with arsenic exposure to accelerate skeletal muscle aging.

Melatonin (MT) is a hormone secreted by the pineal gland that is primarily synthesized and released at night; it plays a critical role in regulating circadian rhythms and regularly receiving a sufficient amount of sleep [13]. Cortisol (COR), a glucocorticoid hormone secreted by the adrenal cortex, plays a key role in metabolic regulation, stress responses, and immune modulation [14]. MT has been shown to promote the regeneration of muscle cells and improve the function of skeletal muscle, particularly during sleep disturbances or when recovering from muscle injury [15]. One clinical study found a negative correlation between the urinary MT metabolite levels and sarcopenia in postmenopausal women, suggesting that MT may protect against muscle loss [16]. On the other hand, elevated COR levels could inhibit the synthesis of muscle proteins and accelerate their breakdown. The prolonged elevation of the COR level not only contributes to muscle atrophy but also increases the risk of aging-associated sarcopenia [17]. Studies have indicated that arsenic exposure could promote the secretion of COR [18], while our previous research demonstrated that chronic exposure to sodium arsenite (iAs) could disrupt the secretion of MT in rats [19]. Therefore, the imbalance of MT and COR secretion could be an essential mechanism for arsenic-induced skeletal muscle aging.

This study aims to determine whether sleep deprivation acts as a risk factor in arsenite-induced skeletal muscle aging, and to investigate its impact on MT and COR secretion. The findings will provide evidence and insights regarding the prevention and treatment of arsenic-induced skeletal muscle damage.

## 2. Materials and Methods

### 2.1. Animals and Treatments

Twenty-four male C57BL/6J mice (8 weeks old; Beijing HFK Bioscience Co., Ltd., Beijing, China) were housed under conventional conditions, at a temperature of 21 °C ± 1 °C and a relative humidity of 50% ± 10%, with a 12 h light/12 h dark cycle (with lights on at 7:00 and lights off at 19:00). After being acclimatized for 1 week, the mice were randomly divided into four groups: the control group (Ctrl), sleep deprivation group (SD), arsenite exposure group (iAs), and arsenite exposure with sleep deprivation group (iAs+SD). All experimental protocols were performed in accordance with and approved by the Animal Experimental Ethical Committee of Guizhou Medical University, Guiyang, China (Approval No. 2303450).

**Figure 1:**
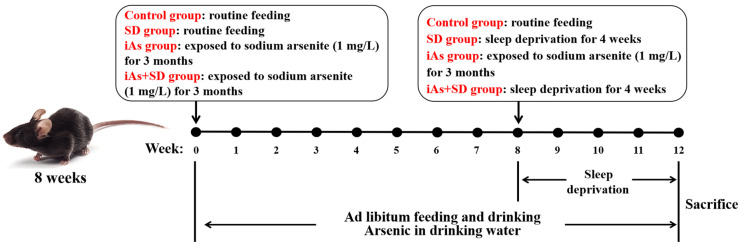
Schematic illustration of the study design.

The mice in the Ctrl group had free access to food and water. The feed was the maintenance feed for mice provided by Huanyu Hio, and the water was deionized water. The mice in the SD group were placed in a sleep deprivation device (ZS-SM-II, ZS Dichuang, Beijing, China), with the platform of the sleep deprivation device moving slowly (parameters: 50 s exercise, 10 s rest, and 10 r/min speed) to ensure that the mice were always awake. The sleep deprivation device was used to place food and water to ensure that the mice could eat and drink normally. The mice in the SD group were deprived of sleep for 20 h (14:00 to next 10:00) every day for 4 weeks [20,21]. This study chose an environmentally relevant dose of arsenite (1 mg/L), based on research indicating that the levels of inorganic arsenic in groundwater frequently range from 0.01 mg/L to 10 mg/L in some countries and regions [22,23]. The sodium arsenite was purchased from Sigma-Aldrich (Shanghai) Trading Co., Ltd., with a purity of no less than 90%. In addition, 1 mg of the sodium arsenite standard was diluted with deionized water to 1 mg/L, and this was freshly prepared for each utilization. The mice in the iAs group were given 1 mg/L of sodium arsenite solution freely for 12 weeks. The mice in the iAs+SD group were given 1 mg/L of sodium arsenite solution freely for 8 weeks and then deprived of sleep and allowed to drink sodium arsenite solution freely for 4 weeks (Figure 1). At the end of the experiment, the mice were sedated via the intraperitoneal injection of pentobarbital sodium, and then they were anesthetized for the collection of cardiac blood.

### 2.2. Determination of Total Arsenic

The method used to pretreat the samples was as follows: the water in the skeletal muscle was adsorbed using filter paper, and 30 mg of skeletal muscle was placed in a conical flask. Then, 5 mL of nitric acid and 2 mL of hydrogen peroxide were added, left for 2 h, and then heated on an electric heating plate until the solution became inert. The color was clear and the acid white smoke evaporated. The sample was concentrated to approximately 1 mL and then transferred and adjusted to a volume of 10 mL with 2% nitric acid. The samples were then shaken, and the sample concentration was determined using an inductively coupled plasma mass spectrometer (ICP-MS, NexION 2000, PerkinElmer, Waltham, MA, USA). The standard curve solution, internal standard solution, and blank control solution (2% nitric acid) were prepared before detection. The detection mode of the instrument was set to collision mode. The arsenic content of the skeletal muscle was calculated using the standard curve and skeletal muscle pretreatment steps.

### 2.3. Limb Grip Strength Testing

The metal mesh of the paw force tester was disinfected and cleaned with 75% ethanol before testing. The instrument was set to zero and the tails of the mice were suspended to ensure that the mice were grasping the metal mesh attached to the dynamometer. Then, the tails of the mice were gently pulled to keep their body parallel to the mesh until the mice released the mesh. The maximum limb grip strength was recorded for each measurement, five times per mouse, and the average value was calculated.

### 2.4. Calculation of the Muscle Index

The muscle index is employed to determine the proportion or status of muscle mass in relation to an individual’s body size or total body mass. It is frequently utilized to evaluate muscle health or sarcopenia, especially in the context of aging or diseases. The mice were therefore euthanized after being weighed, and the gastrocnemius muscle tissue was removed and weighed. The muscle index was calculated according to the following formula: muscle mass (mg)/body mass (g).

### 2.5. Hematoxylin–Eosin (HE) Staining

The fixed tissue wax block was used to perform routine paraffin embedding and sectioning, with the slides then baked at 65 °C for 2 h. The procedure for this was performed as follows: xylene I for 10 min, xylene II for 10 min, absolute ethanol I for 5 min, absolute ethanol II for 5 min, 95% ethanol for 5 min, 90% ethanol for 5 min, and 80% ethanol for 5 min. The block was then rinsed with tap water for 2 min. HE staining was then performed via the following method: The slides were stained with hematoxylin for 10 min, rinsed with tap water for 1 min, differentiated with 1% hydrochloric acid in alcohol for 5 s, and rinsed with tap water for 2 min. Ammonia alcohol was used to blue the slides for 5 s, which were then rinsed with tap water for 1 min, stained with eosin for 5 min, and rinsed with tap water for 30 s. Following this, dehydration and clearing were performed via the following steps: 80% ethanol for 20 s, 90% ethanol for 20 s, 95% ethanol I for 3 min, 95% ethanol II for 3 min, absolute ethanol I for 5 min, absolute ethanol II for 5 min, xylene I for 5 min, and xylene II for 10 min. The slides were then mounted with neutral balsam. Images were observed and captured for analysis using an optical microscope.

### 2.6. Immunofluorescence Staining

The sections were sequentially immersed in solutions of xylene, absolute ethanol, 95% alcohol, 85% alcohol, and 75% alcohol, and rinsed with distilled water. The tissue sections were placed in a pressure cooker containing citric acid (PH 6.0) antigen repair solution for antigen repair, air-jet timing, and natural cooling. The slides were then washed in PBS (PH 7.4). The cells were inactivated using 0.3% methanol hydrogen peroxide and then washed with PBS. A histochemical pen was used to draw a circle around the tissue to prevent reagent spillage. Blocking treatments were performed by adding 5% BSA or 10% goat serum droppers to the pens. The blocking solution was gently removed, the primary antibody that had been prepared in a certain proportion was added to the sections, and the sections were placed flat in a wet box and incubated overnight at 4 °C. Once the slides had been washed in PBS, fluorescent secondary antibodies that corresponded to the species were added to the circles. Then, the tissues were covered and incubated at room temperature in the dark. The slides were washed again and prepared for the next step. DAPI was added drop-wise for the nucleating treatment and washed in PBS. The sections were observed under a fluorescence microscope and images were collected.

### 2.7. Enzyme-Linked Immunosorbent Assay (ELISA)

The serum levels of MT and COR were measured using enzyme-linked immunosorbent assay kits according to the manufacturer’s instructions. The serum levels of MT and COR were measured using Chinese kits produced by Wuhan Fine Biotechnology Co., Ltd (Wuhan, China). The sensitivity of MTn was 4.688 pg/mL, and the sensitivity of COR was 0.234 ng/mL. The coefficients of variation for the intra- and inter-plate were less than 10%. In this kit, the OD value was measured at a wavelength of 450 nm via competitive ELISA, and the MT and COR concentrations were calculated using standard curves.

### 2.8. Quantitative Real-Time Polymerase Chain Reaction (QPCR)

Genomic DNA was extracted from the gastrocnemius muscle using the TIANamp Genomic DNA Kit (Tiangen, Beijing, China). The DNA concentration was quantified using a microplate reader. Samples were adjusted to a final concentration of 5 ng/µL so that their telomere length could be measured. Quantitative PCR (qPCR) was performed using SuperReal PreMix (SYBR Green, Tiangen, Beijing, China). The primers used were as follows: forward TEL 5′-CGGTTTGTTTGGGTTTGGGTTTGGGTTTGGG-3′, reverse TEL 5′-GGCTTGCCTTACCCTTACCCTTACCTTACCC-3′, forward 36B4 5′-ACTGGTCTAGGACCCGAGAAG-3′, and reverse 36B4 5′-TCAATGGTGCCTCTGGAGATT-3′. The relative telomere length was determined by comparing the ratio of the telomere repeat copy number (T) to the single-gene copy number (S, represented by 36B4), which was expressed as the T/S ratio. Each qPCR value was processed using the formula T/S = 2^−∆Ct^, where ∆CT = CT_telomere_ − CT_36B4_. The resulting ratios were then normalized against the reference DNA. All DNA samples were measured in duplicate [24,25].

### 2.9. Oxidative Stress Index Detection

The expression levels of SOD, MDA, and GPx in skeletal muscle were detected via a biochemical method, and all were detected using kits produced by the Nanjing Jiancheng Bioengineering Institute. The skeletal muscle tissue homogenate was prepared as follows: The tissue frozen at −80 °C was taken out, and 30 mg samples of each group were weighed and added to 270 μL of normal saline in an ice water bath to create a 10% skeletal muscle homogenate. The samples were then centrifuged at 3000 rpm for 10 min at 4 °C, and the supernatant, namely 10% homogenate supernatant, was used for testing. Detection was performed according to the instructions, and after the absorbance had been measured, the expression levels of SOD, MDA, and GPx were calculated according to the formula.

### 2.10. Statistical Analyses

Statistical analyses were performed using SPSS 21.0 statistical software (SPSS, Inc., Chicago, IL, USA), and data are expressed as the mean ± standard. Differences between groups were analyzed using one-way ANOVA followed by Tukey’s test. The differences were considered statistically significant at *p* < 0.05.

## 3. Results

### 3.1. The Effect of Sleep Deprivation and Arsenite Exposure on Limb Grip Strength in Mice

**Figure 2:**
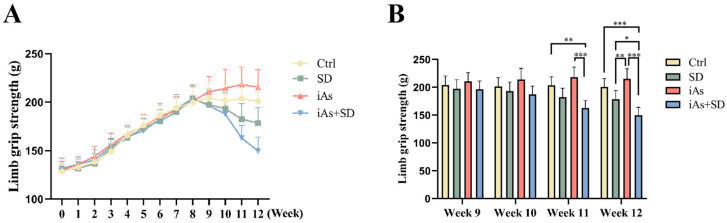
Impact of 1 mg/L arsenite exposure for 12 weeks and/or sleep deprivation for 4 weeks on limb grip strength in mice. Dynamic monitoring of mouse limb grip strength for 12 consecutive weeks (**A**) and the mouse limb grip strength from Week 9 to Week 12 (**B**). Ctrl, control; SD, sleep deprivation; iAs, arsenite; iAs+SD, arsenite exposure with sleep deprivation. The data are expressed as mean ± SD for six mice per group. * *p* < 0.05, ** *p* < 0.01, *** *p*< 0.001.

In order to determine whether sleep deprivation acts as a risk factor in arsenite-induced skeletal muscle aging, the limb grip strength of the mice in each experimental group was dynamically monitored. Before the 10th week, there was no significant difference in limb grip strength between the four groups of mice (Figure 2A). The limb grip strength of the mice in the iAs+SD group began to decline by Week 11 and had significantly decreased by Week 12 compared to the mice in the other groups (Figure 2B).

### 3.2. The Impact of Sleep Deprivation and Arsenite Exposure on Arsenic Concentration and Mass of Skeletal Muscle

**Figure 3:**
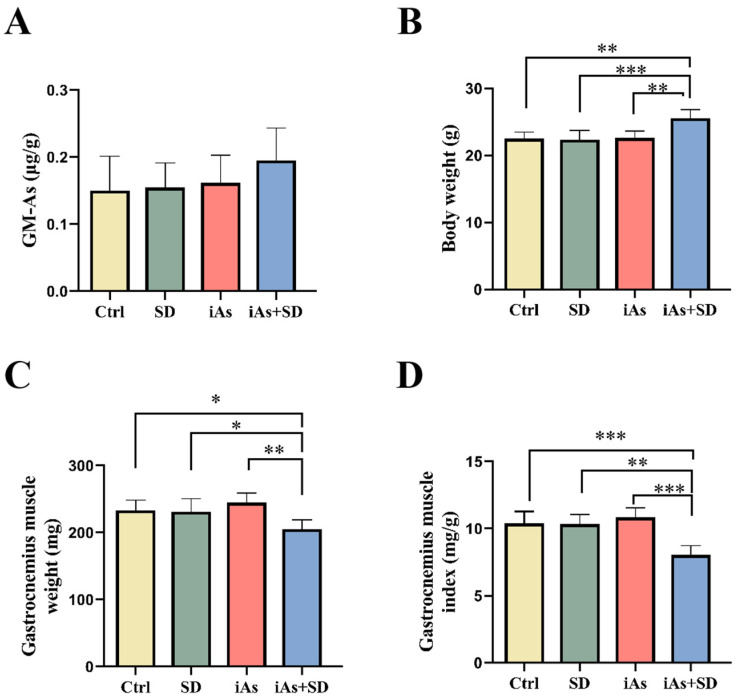
Influence of 1 mg/L arsenite exposure for 12 weeks and/or sleep deprivation for 4 weeks on the arsenic concentrations in the gastrocnemius muscle (**A**), body mass (**B**), gastrocnemius muscle mass (**C**), and gastrocnemius muscle index (**D**) in mice. GM-As, gastrocnemius muscle arsenic concentration; Ctrl, control; SD, sleep deprivation; iAs, arsenite; iAs+SD, arsenite exposure with sleep deprivation. The data are expressed as mean ± SD for six mice per group. * *p* < 0.05, ** *p* < 0.01, *** *p* < 0.001.

At the end of Week 12, the arsenic concentration and skeletal muscle mass were determined. There was no significant difference in the arsenic content of the skeletal muscle of the experimental groups (Figure 3A). Interestingly, the body mass of mice in the iAs+SD group increased significantly compared to that of the other groups (Figure 3B). However, the gastrocnemius muscle mass of mice in the iAs+SD group was significantly lower than that in the other groups (Figure 3C). Compared to the other groups, the muscle index of the iAs+SD group was significantly reduced (Figure 3D).

### 3.3. The Effect of Sleep Deprivation and Arsenite Exposure on Pathological Morphology and Telomere Length of Skeletal Muscle in Mice

**Figure 4:**
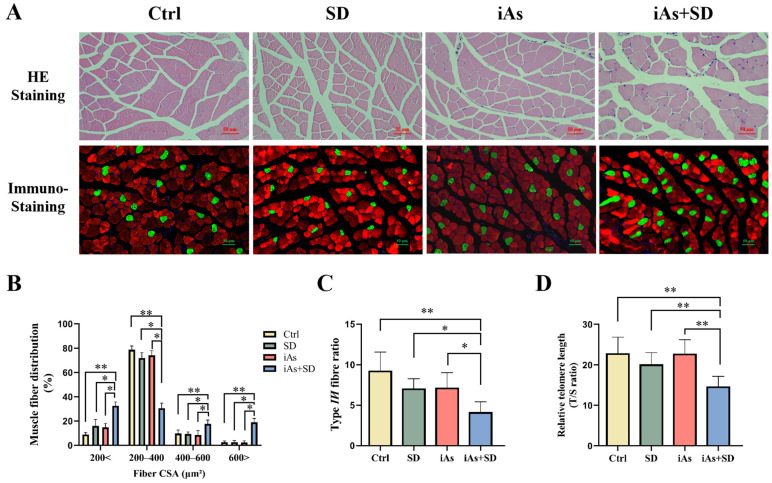
Influence of 1 mg/L arsenite exposure for 12 weeks and/or sleep deprivation for 4 weeks on pathological morphology of the gastrocnemius muscle (**A**), myofiber-type switching (**A**), gastrocnemius muscle fiber cross-sectional area (**B**), type II/I myofiber ratio (**C**), and relative telomere length of the gastrocnemius muscle (**D**) in mice. The green represents type I (slow-twitch) skeletal muscle fiber, and the red represents type II (fast-twitch) skeletal muscle fiber. Scale bars represent 50 µm. Ctrl, control; SD, sleep deprivation; iAs, arsenite; iAs+SD, arsenite exposure with sleep deprivation. The data are expressed as mean ± SD for six mice per group. * *p* < 0.05, ** *p* < 0.01.

The HE staining results showed that the muscle fibers of the Ctrl group were regularly arranged, intact, and clearly delineated, with no observable atrophy, hypertrophy, or necrosis (Figure 4A). There was no significant atrophy of gastrocnemius fibers in the SD and iAs groups compared with the Ctrl group; in addition, some of the gastrocnemius fibers of mice in the iAs+SD group became clearly atrophied, while a few fibers became hypertrophied compared with the other groups (Figure 4A). The cross-sectional area (CSA) of muscle fibers that were less than 200 μm^2^ was significantly increased in the iAs+SD group compared with the other groups (Figure 4B). To further evaluate the impact of arsenic on muscle fiber switching, we conducted immunofluorescence staining for both fast-twitch (type II myofiber) and slow-twitch muscle fibers (type I myofiber) in skeletal muscle tissue. The immunostaining results showed that the number of type I myofibers was increased (Figure 4A), and that the ratio of type II/I myofibers was decreased significantly in the iAs+SD group compared to the other groups (Figure 4C). In order to further demonstrate the impact of arsenic exposure and sleep deprivation on skeletal muscle aging, the relative telomere length of the gastrocnemius muscle was measured. The results showed that the relative telomere length was shortened in the iAs+SD group compared to the mice in other groups (Figure 4D).

### 3.4. The Impact of Sleep Deprivation and Arsenite Exposure on Oxidative Damage in Mice

**Figure 5:**
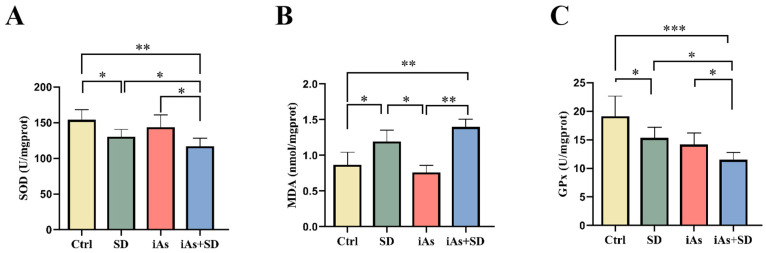
Impact of 1 mg/L arsenite exposure for 12 weeks and/or sleep deprivation for 4 weeks on the activity of SOD (**A**) and the levels of MDA (**B**) and GPx (**C**) in mice. Ctrl, control; SD, sleep deprivation; iAs, arsenite; iAs+SD, arsenite exposure with sleep deprivation. The data are expressed as mean ± SD for six mice per group. * *p* < 0.05, ** *p* < 0.01, *** *p*< 0.001.

Compared with the Ctrl group, the SOD activity and the GPx level in skeletal muscle were reduced; meanwhile, the MDA level was elevated in the SD group (Figure 5A–C). Compared to the mice in the other three groups, the SOD activity and the GPx level of skeletal muscle were significantly reduced in the iAs+SD group (Figure 5A,C); meanwhile, the MDA level was elevated in the iAs+SD group compared with the Ctrl group and iAs group (Figure 5B).

### 3.5. The Impact of Sleep Deprivation and Arsenite Exposure on Serum Levels of MT and COR in Mice

**Figure 6:**
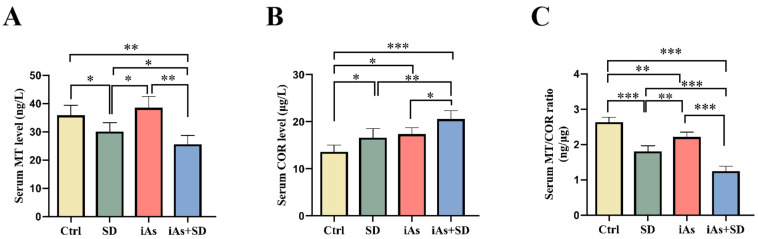
Impact of 1 mg/L arsenite exposure for 12 weeks and/or sleep deprivation for 4 weeks on the levels of serum MT (**A**) and COR (**B**), as well as the ratio of serum MT to COR (**C**) in mice. MT, melatonin; COR, cortisol; Ctrl, control; SD, sleep deprivation; iAs, arsenite; iAs+SD, arsenite exposure with sleep deprivation. The data are expressed as mean ± SD for six mice per group. * *p* < 0.05, ** *p* < 0.01, *** *p*< 0.001.

Compared to the Ctrl group, mice in both the SD and iAs+SD groups exhibited a significant reduction in their serum MT levels (Figure 6A). Conversely, the serum COR levels were upregulated in the SD and iAs groups compared to the Ctrl group, and the iAs+SD group showed significantly higher COR levels compared to the other three groups (Figure 6B). Additionally, the serum MT/COR ratio was decreased in the SD and iAs groups, while the serum MT/COR ratio was significantly decreased in the iAs+SD group relative to the other three groups (Figure 6C).

**Figure 7:**
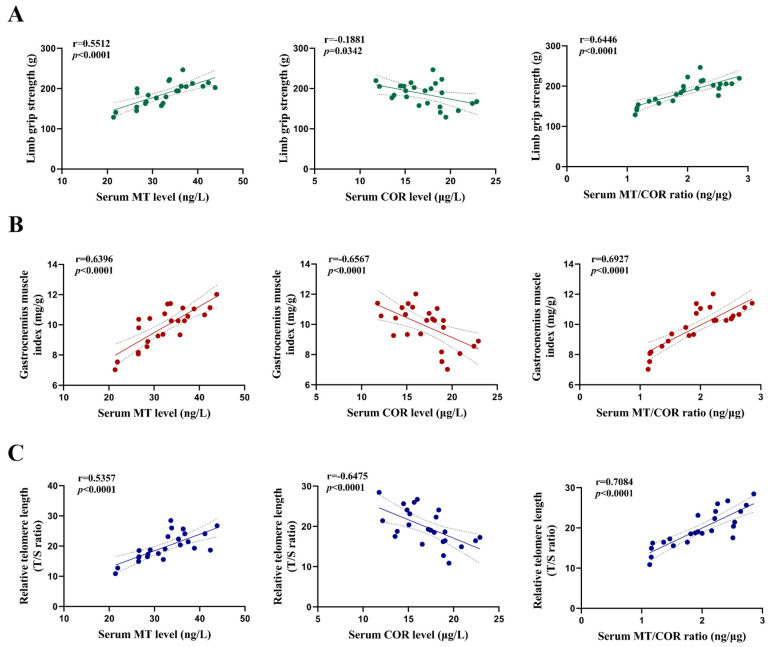
Correlations between serum MT and COR, as well as the ratio of serum MT to COR with limb grip strength, the gastrocnemius muscle index, and relative telomere length of the gastrocnemius muscle in mice. (**A**) The limb grip strength was positively correlated with MT (r = 0.5512, *p* < 0.0001), negatively correlated with COR (r = −0.1881, *p* = 0.0342), and positively correlated with the MT/COR ratio (r = 0.6446, *p* < 0.0001). (**B**) The gastrocnemius muscle index was positively correlated with MT (r = 0.6396, *p* < 0.0001), negatively correlated with COR (r = −0.6567, *p* < 0.0001), and positively correlated with the MT/COR ratio (r = 0.8030, *p* < 0.0001). (**C**) The relative telomere length of gastrocnemius muscle was positively correlated with MT (r = 0.5357, *p* < 0.0001), negatively correlated with COR (r = −0.6475, *p* < 0.0001), and positively correlated with the MT/COR ratio (r = 0.7084, *p* < 0.0001).

The correlation analysis showed that the serum MT level and the ratio of serum MT to COR were positively correlated with limb grip strength, the gastrocnemius muscle index, and relative telomere length of the gastrocnemius muscle; meanwhile, the serum COR level was negatively correlated with the limb grip strength, gastrocnemius muscle index, and relative telomere length of the gastrocnemius muscle (Figure 7A–C).

## 4. Discussion

Arsenic is an environmental endocrine disrupter chemical, and its toxicity to skeletal muscle has been gradually recognized. Previous studies have found that chronic exposure to arsenite is capable of inducing skeletal muscle atrophy and interfering with the secretion of melatonin (MT) in rats. This study provided novel evidence regarding the impact of arsenic exposure on skeletal muscle aging in mice and revealed that sleep loss acts as an important risk factor. In addition, the imbalance between MT and COR secretion may be implicated in the development of sleep loss- and arsenite-induced skeletal muscle aging.

Skeletal muscle aging refers to the progressive loss of skeletal muscle mass and the progressive decline in skeletal muscle function with advancing age; this negatively affects a variety of physiological parameters, including vision, breathing, and movement [26]. Recent epidemiological studies have indicated the association between arsenic exposure and the loss of skeletal muscle mass among individuals living in areas of Bangladesh with high levels of arsenic [4,5]. We previously revealed the effects of arsenite exposure on skeletal muscle mass in rats [19], and this study further investigated the effects of arsenite exposure on skeletal muscle mass and function, which was often reflected by limb grip strength [27]. We dynamically monitored the changes in the skeletal muscle function of mice via limb grip strength testing and found that the limb grip strength of mice in the iAs+SD group began to decline at Week 11 and had significantly decreased by Week 12 compared to the mice in the other groups. At the end of Week 12, we also found that other phenotypes of skeletal muscle aging were more significant in the iAs+SD group than in the other groups; these phenotypes included the loss of gastrocnemius muscle mass and atrophy, an increased proportion of type II muscle fibers, the shortening of the telomere length, and an increased level of oxidative damage. These studies suggested that arsenite exposure could induce multiple phenotypes of skeletal muscle aging.

It is well-known that long-term exposure to arsenic can induce multiple-organ damage, and growing evidence has indicated that arsenic exposure is an environmental risk factor for skeletal muscle damage. However, the factors that are implicated in arsenic-induced skeletal muscle damage remain unclear. Therefore, this research focused on whether there are factors present in environmental arsenic-contaminated areas that can synergize with arsenic exposure to accelerate the loss of skeletal muscle. Previously, we revealed that chronic exposure to arsenite could induce skeletal muscle atrophy in rats [19]. However, considering that skeletal muscle is characterized by age-related decline, we also investigated whether arsenite-induced skeletal muscle damage in rats is age-related. We further found that subchronic exposure to arsenite could induce skeletal muscle atrophy and the shortening of telomeres in middle-aged rats (12 months old), but that it has no significant effect on young rats (3 months old) [28]. These results suggest that age is one of the key factors affecting arsenite toxicity in skeletal muscle. In addition, sleep deprivation could reduce the synthesis of proteins in skeletal muscle [12], which is another key factor leading to the loss of skeletal muscle mass. Therefore, we further explored whether sleep deprivation could synergize with arsenite exposure to accelerate skeletal muscle aging. The results showed that sleep deprivation for 4 weeks could induce multiple phenotypes of skeletal muscle aging after subchronic exposure to arsenite in mice. In addition, we found that some of the muscle fibers became hypertrophic in the iAs+SD group, which may be a compensatory response. These studies suggested that sleep deprivation may increase the sensitivity of arsenite exposure to skeletal muscle aging and lower its threshold.

**Figure 8:**
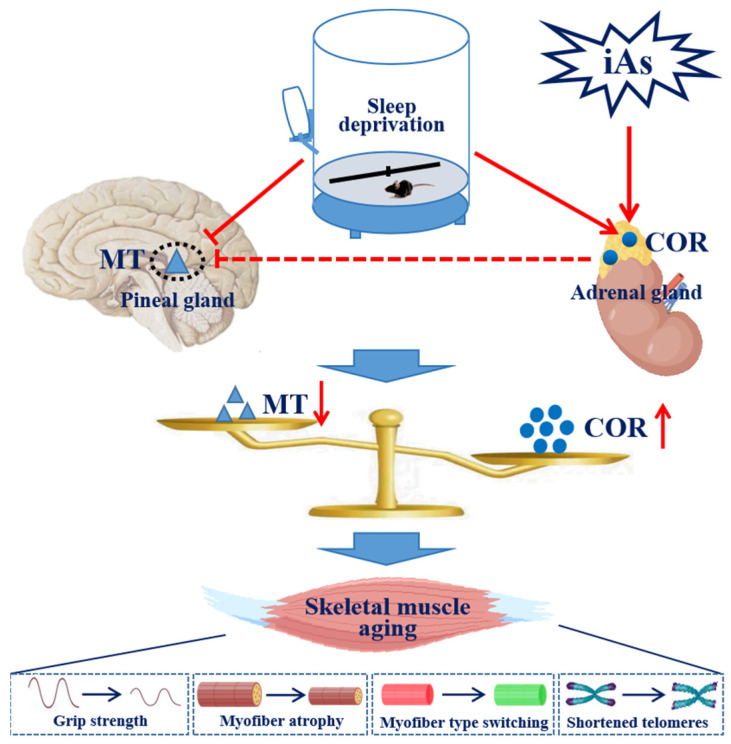
Proposed model of the impact of sleep deprivation and the synergistically toxic effect of subchronic arsenite exposure on skeletal muscle in mice. Sleep deprivation and subchronic arsenite exposure synergistically induced skeletal muscle aging in mice, together with an imbalance between MT and COR secretion.

MT and COR play a significant role in maintaining skeletal muscle mass. Therefore, MT could be employed in the prevention and treatment of sarcopenia [29]; it has also been found that the loss of skeletal muscle mass and function is a typical clinical manifestation of patients with hypercortisolism [30,31]. We previously constructed a rat model of arsenite-induced skeletal muscle damage and found that long-term exposure to arsenite could suppress the secretion of MT [28]. Moreover, epidemiological studies have shown that maternal prenatal inorganic arsenic exposure is associated with elevated levels of salivary COR in infants, and that long-term exposure to arsenite may trigger abnormally elevated levels of COR by activating the hypothalamic–pituitary–adrenal (HPA) axis [18,32]. These results showed that arsenite could increase the serum COR level in mice. Importantly, studies have shown that a lack of sleep could disrupt the secretion of MT and COR [33]. The results suggested that sleep deprivation and arsenic exposure could synergistically inhibit the serum MT level and increase the serum COR level, indicating that an imbalance in MT and COR secretion could be involved in the synergistic effect of sleep deprivation and arsenite exposure on skeletal muscle aging (Figure 8).

Studies have shown that oral MT could effectively reduce the urinary COR level in patients with fibromyalgia [34]. The level of serum MT was significantly decreased and the secretion of COR was significantly increased in patients undergoing pinealectomy [35]. In addition, the secretion of MT decreased in Cushing’s syndrome patients [36]. Metyrapone (a COR inhibitor) could inhibit the secretion of COR and increase the serum MT level in patients with hyperthyroidism [37]. These studies suggested that there may be a negative regulatory relationship between MT and COR. Interestingly, studies have shown that the ratio of MT/COR is associated with metabolic syndrome, endocrine disorders, and depression [38,39]. Therefore, we further analyzed the ratio of serum MT to COR and various indicators of skeletal muscle aging, including the skeletal muscle mass index, telomere length, and limb grip strength. The results showed that the correlations between the serum MT/COR ratio and skeletal muscle aging indicators were more significant than those between the serum MT or COR level alone, suggesting the ratio of serum MT to COR could more accurately reflect their homeostasis.

## 5. Conclusions

In conclusion, the results of this study indicate that prolonged sleep deprivation for 4 weeks may lower the threshold for skeletal muscle toxicity induced by subchronic exposure to 1 mg/L arsenite, and that the imbalance of MT and COR secretion may be involved in the synergistic effect of sleep deprivation and arsenite exposure on skeletal muscle aging. Further studies on the dual regulation of MT and COR under arsenite exposure is of great significance for the prevention and treatment of arsenite-induced skeletal muscle damage. However, further studies should focus on the toxic effects of pentavalent and organic arsenic on skeletal muscle, and determine whether the phenotypes of skeletal muscle aging in arsenic-exposed populations are associated with the abnormal secretion of MT and COR.

## Data Availability

The data presented in this study are available upon request from the corresponding author.
